# Genome-wide small RNA profiling reveals tiller development in tall fescue (*Festuca arundinacea* Schreb)

**DOI:** 10.1186/s12864-020-07103-x

**Published:** 2020-10-06

**Authors:** Tao Hu, Tao Wang, Huiying Li, Misganaw Wassie, Huawei Xu, Liang Chen

**Affiliations:** 1 CAS Key Laboratory of Plant Germplasm Enhancement and Specialty Agriculture, Wuhan Botanical Garden, The Innovative Academy of Seed Design, Chinese Academy of Sciences, Wuhan, 430074 China; 2 Center of Economic Botany, Core Botanical Gardens, Chinese Academy of Sciences, Wuhan, 430074 China; 3 College of Agriculture, Henan University of Science and Technology, Luoyang, China; 4 University of Chinese Academy of Sciences, Beijing, 100049 China

**Keywords:** MicroRNAs, Tiller development, Small RNA deep sequencing, Stem-loop qRT-PCR, Tall fescue

## Abstract

**Background:**

Tall fescue (*Festuca arundinacea* Schreb.) is a major cool-season forage and turfgrass species. The low tiller density and size dramatically limits its turf performance and forage yield. MicroRNAs (miRNA)-genes modules play critical roles in tiller development in plants. In this study, a genome-wide small RNA profiling was carried out in two tall fescue genotypes contrasting for tillering production (‘Ch-3’, high tiller production rate and ‘Ch-5’, low tiller production rate) and two types of tissue samples at different tillering development stage (Pre-tillering, grass before tillering; Tillering, grass after tillering). ‘Ch-3’, ‘Ch-5’, Pre-tillering, and Tillering samples were analyzed using high-throughput RNA sequencing.

**Results:**

A total of 222 million high-quality clean reads were generated and 208 miRNAs were discovered, including 148 known miRNAs belonging to 70 families and 60 novel ones. Furthermore, 18 miRNAs were involved in tall fescue tiller development process. Among them, 14 miRNAs displayed increased abundance in both Ch-3 and Tillering plants compared with that in Ch-5 and Pre-tillering plants and were positive with tillering, while another four miRNAs were negative with tiller development. Out of the three miRNAs osa-miR156a, zma-miR528a-3p and osa-miR444b.2, the rest of 15 miRNAs were newfound and associated with tiller development in plants. Based on our previous full-length transcriptome analysis in tall fescue, 28,927 potential target genes were discovered for all identified miRNAs. Most of the 212 target genes of the 18 miRNAs were dominantly enriched into “ubiquitin-mediated proteolysis”, “phagosome”, “fatty acid biosynthesis”, “oxidative phosphorylation”, and “biosynthesis of unsaturated fatty acids” KEGG pathways. In addition, bdi-miR167e-3p targets two kinase proteins EIF2AK4 and IRAK4, and osa-miR397a targets auxin response factor 5, which may be the significant miRNA-genes controllers in tillering development.

**Conclusions:**

This is the first genome-wide miRNA profiles analysis to identify regulators involved in tiller development in cool-season turfgrass. Tillering related 18 miRNAs and their 212 target genes provide novel information for the understanding of the molecular mechanisms of miRNA-genes mediated tiller development in cool-season turfgrass.

## Background

Tall fescue (*Festuca arundinacea* Schreb.) is a main cool-season grass species, widely applied as forage and turf for gardens, parks, residential and sports grounds [1, 2]. Because of its agronomic importance, tall fescue is grown commonly in temperate regions of the world including the United States, China, Japan, Australia, and many countries in Europe [1]. However, the low tiller density and size are the major factor limiting its turf performance and dry matter yield of forage.

Tiller number is the most important agronomic trait for cool-season grass and is responsible for high shoot density and biomass production [3, 4]. The morphological observation of tillering development in monocot grass showed that tiller production is normally formed in two distinct developmental stages including axillary bud formation and its subsequent outgrowth or extension [4, 5]. A tiller axillary bud is formed from the tiller primordium and extends into primary tillers, which then induces a new tiller axillary bud that develops into secondary tillers. Therefore, the grass develops more tiller number with more tiller axillary buds. Under optimal growth environment, tall fescue develops tertiary, quartus and more tillers. It is well reported that tillering is a complex trait that can be regulated by multiple factors such as endogenous hormones level, water, fertilizer, light, temperature, genes, and miRNA [6, 7].

MicroRNAs (miRNAs) are a category of approximately 20–24 nucleotides (nt) long small endogenous non-coding small RNAs (sRNAs), that directly involved in the suppression of target genes [8]. The biogenesis of plant miRNAs begins with the transcription of miRNA genes (MIRs) which usually located in the intergenic regions and has promoter. MIRs are transcribed into a stem-loop structured precursor called miRNAs (pri-miRNAs) by RNA polymerase II (Pol II). pri-miRNAs are processed by Dicer-like protein 1 (DCL1), HYPONASTIC LEAVES1 (HYL1), and SERRATE (SE) to form the miRNA/miRNA* duplexes. Following the pri-miRNA processing, HUA ENHANCER 1 (HEN1) replaces SE to catalyzes 2′-O-methylation at the 3’ends of miRNA duplex, which further is loaded into an ARGONAUTE 1 (AGO1) protein to form an active miRNA-induced silencing complex (miRISC) [9]. AGO1-miRISC performs transcript cleavage of target miRNAs by pairing miRNAs and their target mRNAs [10]. But, translational repression of target genes by AGO1-miRISCs is not depending on the sequence complementarity of miRNA target sites. AGO1-miRISCs blocks ribosome recruitment and translation initiation by binding to the 5′ untranslated regions (UTRs) or 3′ UTRs [11]. A recent study also reported that pri-miRNAs can be processed into long miRNAs (24 nt) and are sorted into the effector AGO4 to direct DNA methylation at the transcriptional level [12].

Studies showed that a single miRNA or one miRNA family often plays an essential role in regulating various aspects of plant development and stress responses through the three miRNA actions including target messenger RNA cleavage, translational repression, and DNA methylation [13, 14].

In plants, miRNAs are evolutionarily conserved and tend to have conserved targets among different plant species. For example, the miR172 and its target AP2/AP2-like could control flowering time in a variety of plant species such as barley, soybean, rice and maize [11]. The miR168 and its target AGO1 are involved in pathogen immune response in rice, tobacco and Arabidopsis, [15]. On the other hand, overexpression of miR395 in Arabidopsis and rice induced S-starvation symptoms [16, 17]. In addition to the conserved functions, some miRNAs have also a diversified regulatory function. For instance, the miR319-TCP module was involved in cold tolerance in sugarcane and participated in drought/salt tolerance in creeping bentgrass [11]. Overexpression of miR393 significantly increased the number of tillers and biomass yield in switchgrass and rice [18, 19].

In rice, tillering is also controlled by miR156, miR172 and miR444 [20–22]. In turfgrass, miR528-overexpressing transgenic plants showed increased tiller number in creeping bentgrass [23]. These results demonstrate that conserved or non-conserved miRNAs were in positive modulation of tillering and could be potentially applied in molecular breeding to enhance the number of grass tiller. However, there is a strong need to identify more miRNAs controlling tiller bud initiation and outgrowth. More importantly, the role of miRNAs in controlling tillering development remains unclear.

Using next-generation high-throughput sequencing technologies, genome-wide sRNAs profiling analysis has identified numerous plant miRNAs. Until now, thousands of miRNAs have been discovered in different plant species such as rice, maize, barley, soybean, etc. [15, 24], which help to determine the multigenic net regulatory mechanisms of tillering development and breeding of ideal plant type. Recently, Li et al. [25] demonstrated a genome-wide sRNAs profiling in two tall fescue genotypes with distinct thermotolerance and identified 850 miRNAs involved in heat stress response. The genome size of the tall fescue is approximately 6 × 10^3^ Mbp, which is about 14 times larger than that of rice. To date, a lot of miRNAs had not been discovered in tall fescue due to the limited data concerning its genome. An accelerated effort to acquire the genome-wide sRNAs profiling of tall fescue in tillering development will be helpful to develop ideal tall fescue genotypes.

Previously, the two tall fescue genotypes, ‘CH-3’ and ‘CH-5’, with high and low tillers production rate were identified from turfgrass germplasm bank in our lab. Vegetative- and tillering-stage tissues of ‘Houndog V’ were named Pre-tillering and Tillering samples, respectively. Coupling analysis of the four groups; Ch-3, Ch-5, Pre-tillering, and Tillering will effectively remove the background interference of genotypic variation and tissue development changes. Tillering-stage tissues of ‘CH-3’, ‘CH-5’, Pre-tillering, and Tillering were subsequently collected for sRNAs sequencing. A total of 222 million clean reads were generated and 208 miRNAs with 28,927 potential target genes were identified. Finally, we newly identified 15 specific miRNAs that have not been reported in other plant species participating in tillering development. The present study provides novel insights into the comprehensive regulation pattern of miRNA-genes controlling tiller development in tall fescue.

## Results

### Tillering characterization of tall fescue

**Fig. 1:**
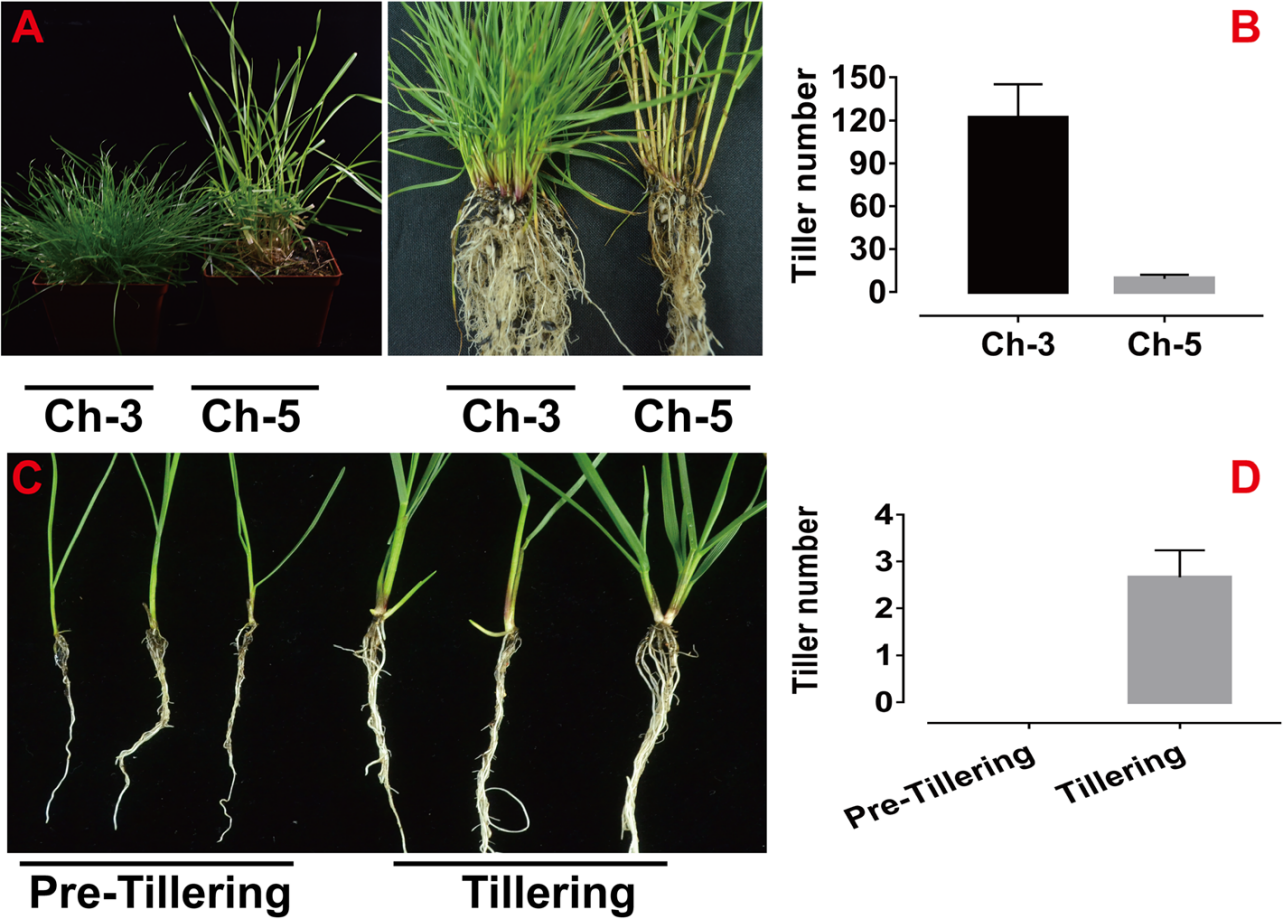
Tillering phenotypes of ‘Ch-3’, ‘Ch-5’, Pre-tillering and Tillering plants. **a** and **b** Tillering phenotypes in ‘Ch-3’ and ‘Ch-5’after 2 months of the establishment. **c** and **d** Tillering phenotypes in ‘Houndog V’ at the 2.5-leaf stage (Pre-tillering) and at the 4.5-leaf stage (Tillering). Each measurement included eleven independent biological repetition. Vertical bars indicated LSD values where significant difference were detected (*P* < 0.05)

The two tall fescue genotypes (Ch-3 and Ch-5) showed a remarkable difference in tillering development (Fig. 1). Ch-3 with high tiller production rate had 122 tillers, but Ch-5 had only 9 tillers after 2 months of the establishment (Fig. 1a, b). Furthermore, we found that Ch-3 plants were still undergoing vegetative growth while Ch-5 plants have begun heading growth (Fig. 1a). Therefore, the difference in tiller number between the two tall fescue genotypes reached its maximum during this growth stage, which was the most appropriate period to collect for sRNAs sequencing. But, the tall fescue ‘Houndog V’ plants showed different growth stage (Fig. 1c and d.) At the 2.5-leaf stage, plants were at the vegetative growth and the tiller number was zero, which named Pre-tillering. However, at the 4.5-leaf stage, plants began to tiller and the tiller number attained two or three, which named Tillering. Hence, in order to identify the miRNAs involved in tillering more accurately, samples were collected both at vegetative growth and tillering stages.

### Deep sequencing of sRNAs

To identify and characterize the role of miRNAs in the tillering development of tall fescue, the samples from two genotypes Ch-3 and Ch-5 with different tillering, and the Pre-tillering and Tillering samples from ‘Houndog V’ at the different tillering development stage were used for sRNAs libraries construction and sequencing. Using the Illumina HiSeq 2500 system, a total of 222 million high-quality clean reads were obtained and each sample produced more than 18 million clean reads averagely. By choosing 18–30 nt clean reads, about 55.97% (5075789), 43.4% (5814258), 47.9% (7516722) and 47.7% (7501722) of the total reads were perfectly mapped to the full-length transcriptome data of tall fescue for Ch-3, Ch-5, Pre-tillering and Tillering, respectively (Additional file 1). Through scanning the size distribution based on total unique reads of the four samples, 24-nt sRNAs were the most abundant in all samples, accounting 36.8, 36.2, 35.33%, and 35.94 in Ch-3, Ch-5, Pre-tillering and Tillering, respectively (Additional file 2). The total mapped reads were grouped into various noncoding sRNA categories, including known miRNA (0.16–0.43%), rRNAs (12.87–24.14%), tRNAs (0%), snRNAs (0.13–0.49%), snoRNAs (0.13–0.25%) and unmatched sRNAs (75.28–84.4%) as shown in Table 1. The total reads that could be annotated to known miRNAs were 9079, 28,516, 23,705, and 28,673 for Ch-3, Ch-5, Tillering, Pre-tillering, respectively. Interestingly, Ch-3 with high tiller production rate showed less number of known miRNAs than that of Ch-5. Consistent with this result, the Tillering samples had fewer known miRNAs compared with the Pre-tillering samples.

**Table 1:** Distribution of unique reads among different categories in tall fescue

Category	Ch-3	Ch-5	Tillering	Pre-tillering
**Total**	**5,075,789 (100%)**	**5,814,258 (100%)**	**7,516,722 (100%)**	**7,501,722 (100%)**
**Known miRNA**	**9079 (0.16%)**	**28,516 (0.43%)**	**23,705 (0.34%)**	**28,673 (0.40%)**
**rRNA**	**1,236,690 (24.14%)**	**1,034,765 (17.95%)**	**1,136,985 (14.87%)**	**1,210,625 (16.10%)**
**tRNA**	**1 (0.00%)**	**1 (0.00%)**	**3 (0.00%)**	**2 (0.00%)**
**snRNA**	**12,537 (0.27%)**	**27,204 (0.49%)**	**10,006 (0.13%)**	**10,312 (0.14%)**
**snoRNA**	**7674 (0.15%)**	**7637 (0.13%)**	**18,656 (0.25%)**	**16,876 (0.22%)**
**Other**	**3,809,809 (75.28%)**	**4,716,135 (81.00%)**	**6,327,366 (84.40%)**	**6,235,234 (83.14%)**

### Identification of known and novel miRNAs

**Fig. 2:**
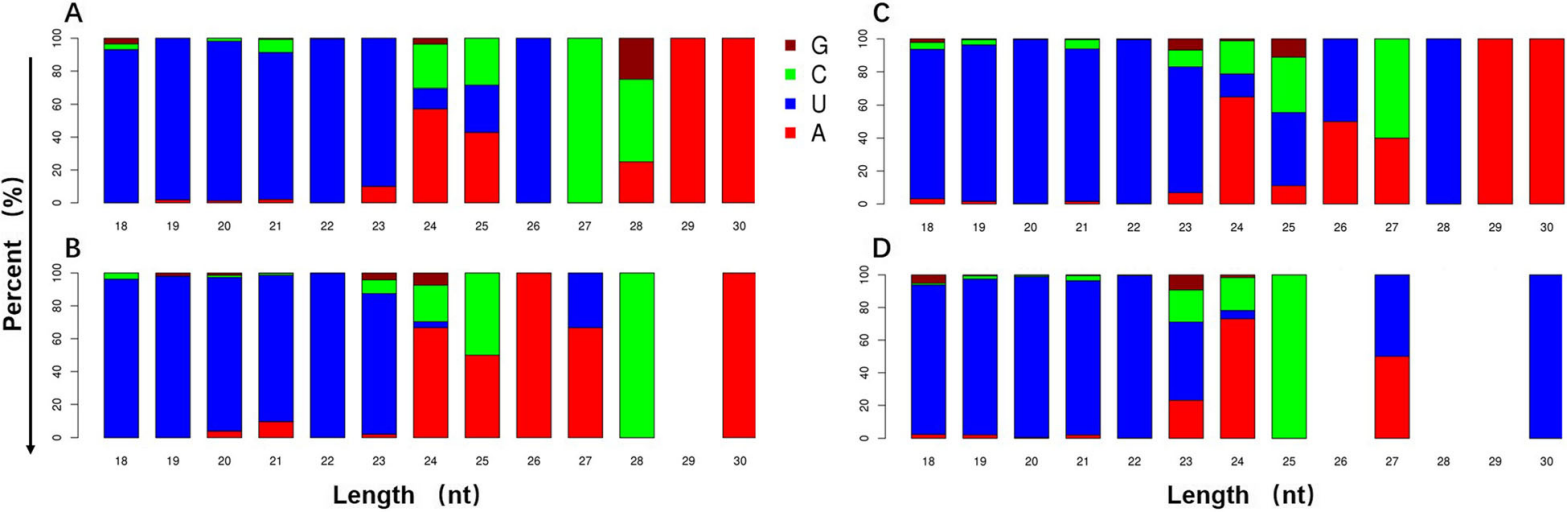
The first base preference of known miRNA mature. 18 ~ 30-nt sRNAs were selected for analyzed and each histogram indicated the percentage of first base in the sRNAs with same RNA number. **a**, **b**, **c** and **d** represents ‘CH3’, ‘CH5’, ‘Tillering’ and ‘Pre-tillering’ samples, respectively

To identify known and novel miRNAs in tall fescue, all the unannotated unique reads that perfectly mapped to tall fescue full-length transcriptome data were aligned to plant miRNAs in miRBase21 database. A total of 3051 miRNA precursors were identified and then compared with mature miRNAs from *Brachypodium distachyon*, *Oryza sativa*, *Zea mays*, *Hordeum vulgare*, *Aegilops tauschii* and *Festuca arundinacea*. Accordingly, 148 known miRNAs belonging to 70 families were identified (Additional files 3, 4). Among them, 60 families were well conserved and found in more than two plant species. Especially, miR395, miR399, miR169, miR166, miR160, miR172 and miR395 were highly conserved and identified in nearly 30 plant species (Additional file 4). Besides, we analyzed the first base preference for known mature miRNA (Fig. 2). Among these four groups: Ch-3, Ch-5, Pre-tillering and Tillering showed preference towards U for the first base in 18 ~ 23-nt sRNAs, but the first base preference in 24 ~ 30-nt sRNAs was different between the four groups. Ch-3 and Tillering had more U preference than Ch-5 and Pre-tillering in 24 ~ 35-nt sRNAs. On the other hand, 10 families such as miR5048, miR5185, miR9863, miR7708, etc. were less conserved and found only in one plant species. In addition to these known miRNAs, 60 novel miRNAs were identified by predicting the hairpin structures of their precursor sequences (Additional file 5), and the abundance of U was decreased for the first base preference in 18 ~ 30-nt sRNAs for the identified novel miRNAs (Additional file 6).

### Differential expression of miRNAs during tillering development

**Fig. 3:**
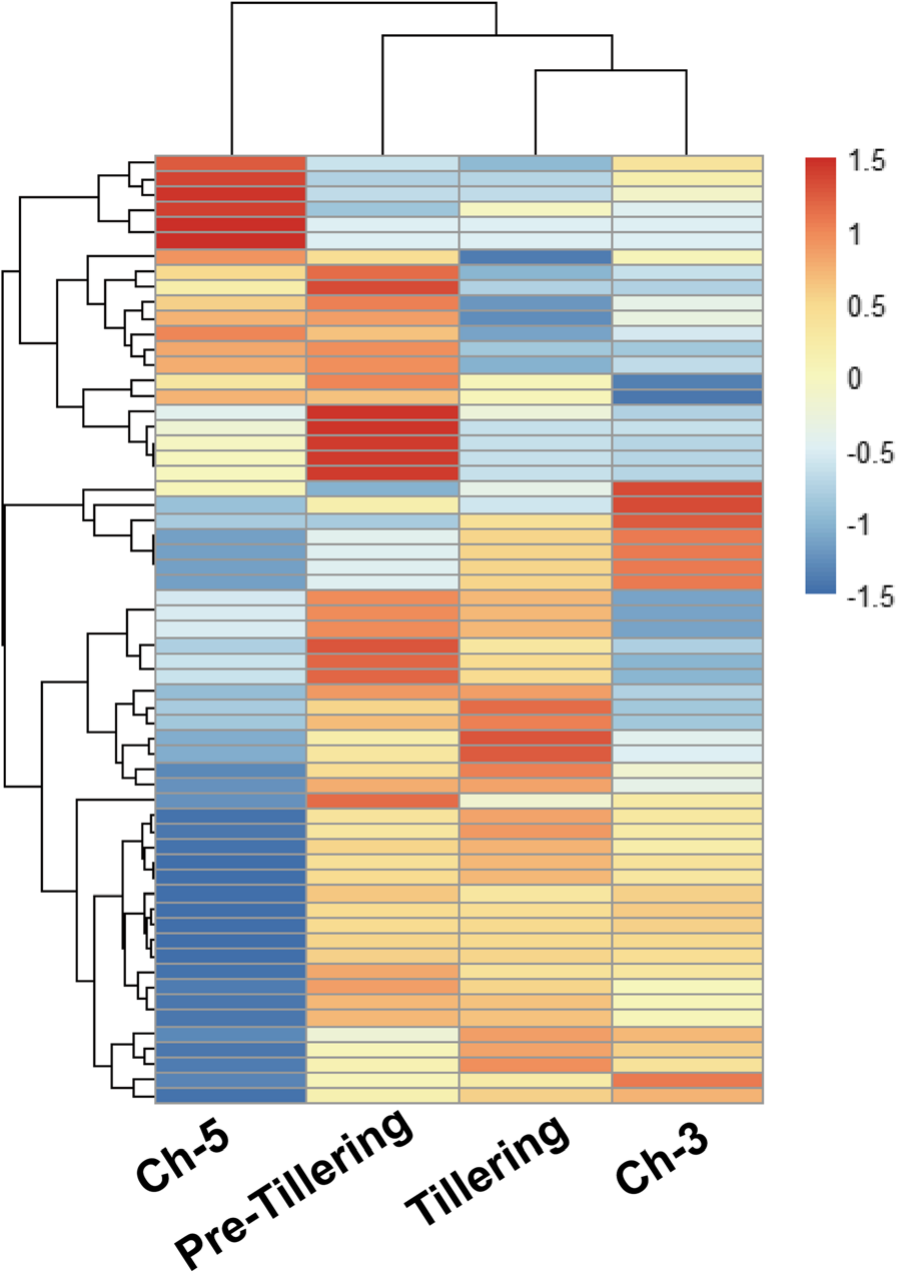
Heatmap of Cluster analysis of the log2TPM of miRNAs. The bar represents the scale of miRNA expression levels of miRNAs. The resulting tree figures were displayed using the software package, Java Treeview. Red, up-regulation; blue, down-regulation

To identify the genome-wide small RNAs involved in tillering development, the different expression levels of known and novel miRNAs were clustered using log10 (TPM + 1). The miRNAs detected in the two genotypes or different tillering development stages were separated (Fig. 3; Additional file 7), indicating that the tiller development was regulated by miRNAs. Based on the read count value, a total of 34 miRNAs from 29 miRNA families exhibited differential accumulation between Ch-3 and Ch-5 genotypes (Additional file 8). We found 24 significantly up-regulated and 10 down-regulated miRNAs in Ch-3 when compared to Ch-5. Furthermore, the abundance of various miRNAs at the tiller development stage was altered. In tillering plants, the expression levels of 16 miRNAs were increased, while 13 miRNAs were decreased compared with the non-tillering plants (Additional file 9).

To identify the key miRNAs controlling tillering in tall fescue, we performed the Venn diagram analysis and measured 14 miRNAs co-up-regulated and 4 miRNAs co-down-regulated at both Ch-3/Ch-5 and Tillering/Pre-tillering groups (Additional file 10). The differentially co-expressed miRNAs were 5 novel and 13 known miRNAs belonging to 15 families (Table 2). Furthermore, in order to check whether the 18 miRNAs is relative to tall fescue tillering, we measured the expression level of six randomly picked miRNAs from them using Stem-Loop qRT-PCR. As shown in Additional file 11, bdi-miR160f, novel_22, novel_23, osa-miR156a, osa-miR408-3p and osa-miR394 showed the same change pattern in Ch3 and Ch5 plants compared with the sequencing data. In addition, novel_22, novel_23, osa-miR156a, osa-miR408-3p and osa-miR394 showed higher transcriptional level in axillary bud than that in out growth bud samples, and they also displayed more expression level in Ch3 than that in Ch5. The bdi-miR160f showed lower transcriptional level in Ch3 than that in Ch5, and it was lower in axillary bud than that in out growth bud samples. The finding indicates that the 18 miRNAs may play key roles in tall fescue tillering development.

**Table 2:**
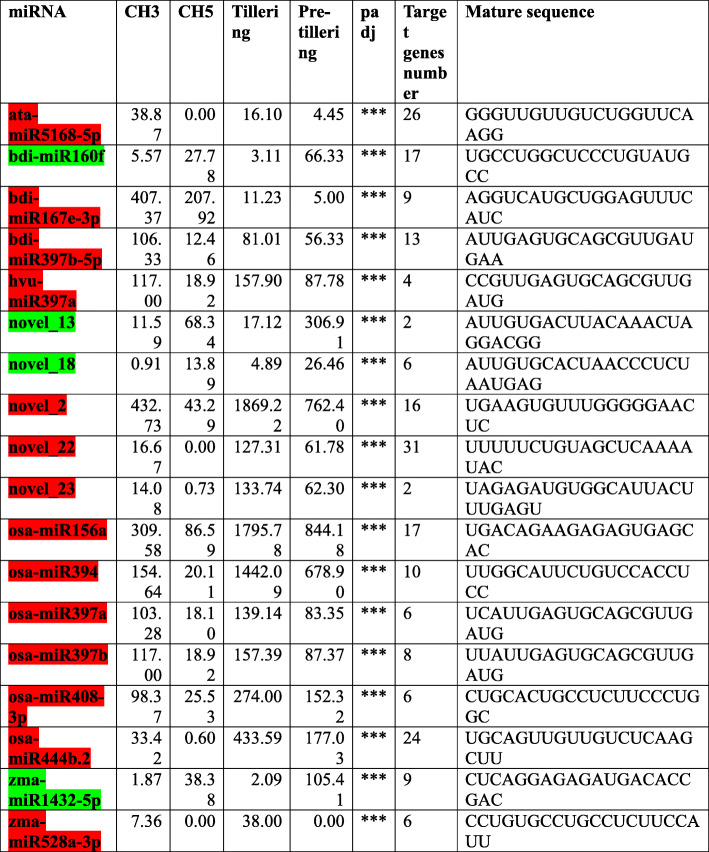
Co-up-regulated or co-down-regulated miRNAs at both Ch-3/Ch-5 and Tillering/Pre-tillering groups

The red indicates the miRNA is positive with tillering, and the green indicates the miRNA is negative with tillering. Transcript abundance of miRNAs in Ch-3, Ch-5, Tillering and Pre-tillering was present with readcount value. *** indicates the padj < 0.0001. The details of target genes is shown in Additional file 10

### Prediction and enrichment of miRNA target genes

**Fig. 4:**
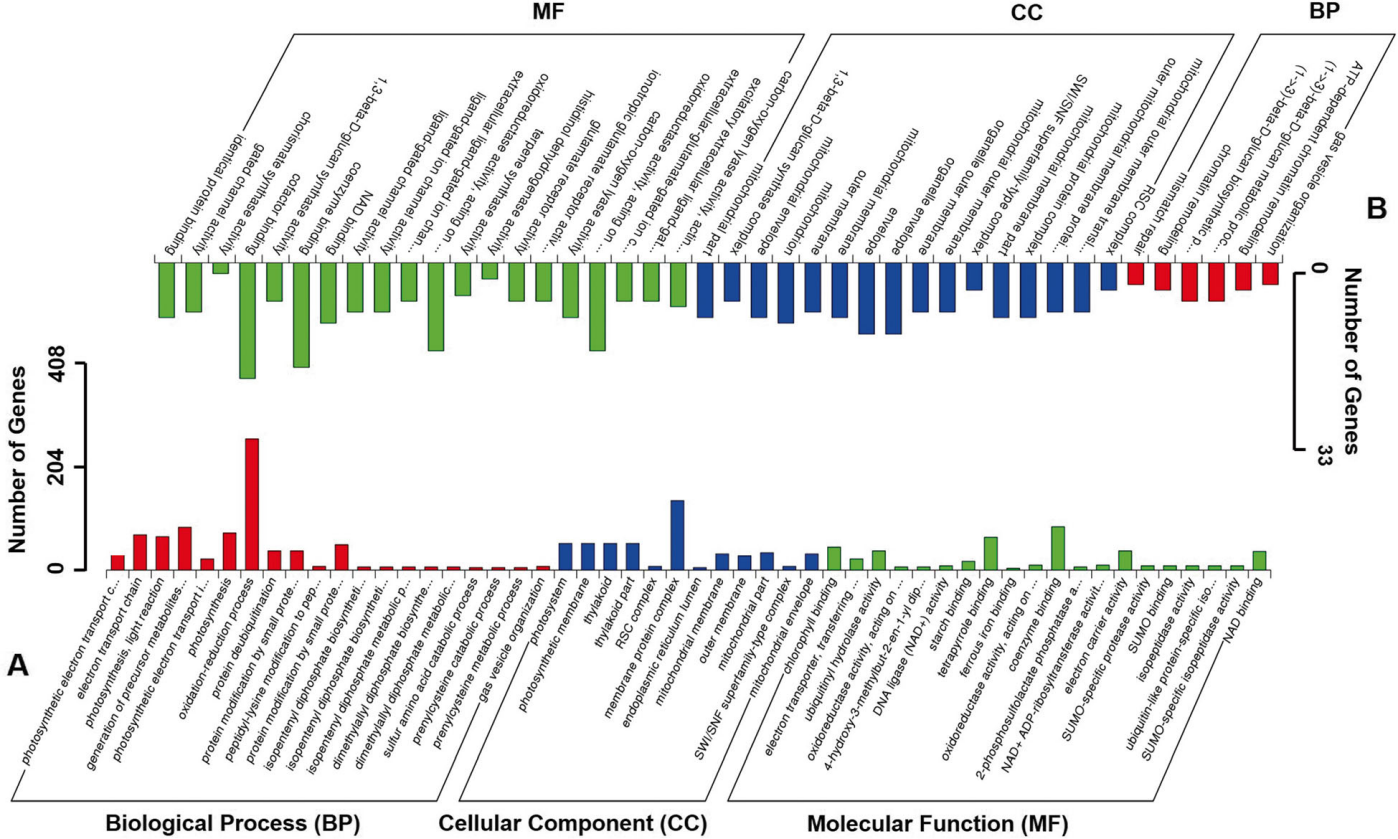
Functional categorization and distribution of miRNA target genes with different expression levels based on Gene Ontology (GO) classification in tall fescue. The target genes were summarized in Biological Process (BP), Cellular Component (CC), and Molecular Function (MF) three main GO categories. **a**, the differentially expressed miRNAs between ‘CH3’ and ‘CH5’ with 53 subcategories. **b**, the differentially expressed miRNAs between ‘Tillering’ and ‘Pre-tillering’ with 43 subcategories

To gain additional insights into the miRNA-genes pathway that related to tiller development in tall fescue, we obtained a total of 28,927 potential target genes for all known and novel miRNAs from Ch-3, Ch-5, Tillering and Pre-tillering samples (Additional file 12). The number of target genes for each miRNA showed a remarkable difference, such as bdi-miR5049-3p and bdi-miR5067 had only one target gene but bdi-miR845 and osa-miR414 targeted more than 30 genes. Here, miRNA regulated targeted genes through two miRNA actions: target messenger RNA cleavage and translational repression. GO enrichment analysis was performed to evaluate the potential functions of target genes of differential expression miRNA. A total of 2093 target genes of miRNA with differential accumulation between Ch-3 and Ch-5 were identified and categorized into 52 GO functional subcategories (Fig. 4; Additional file 13).

**Fig. 5:**
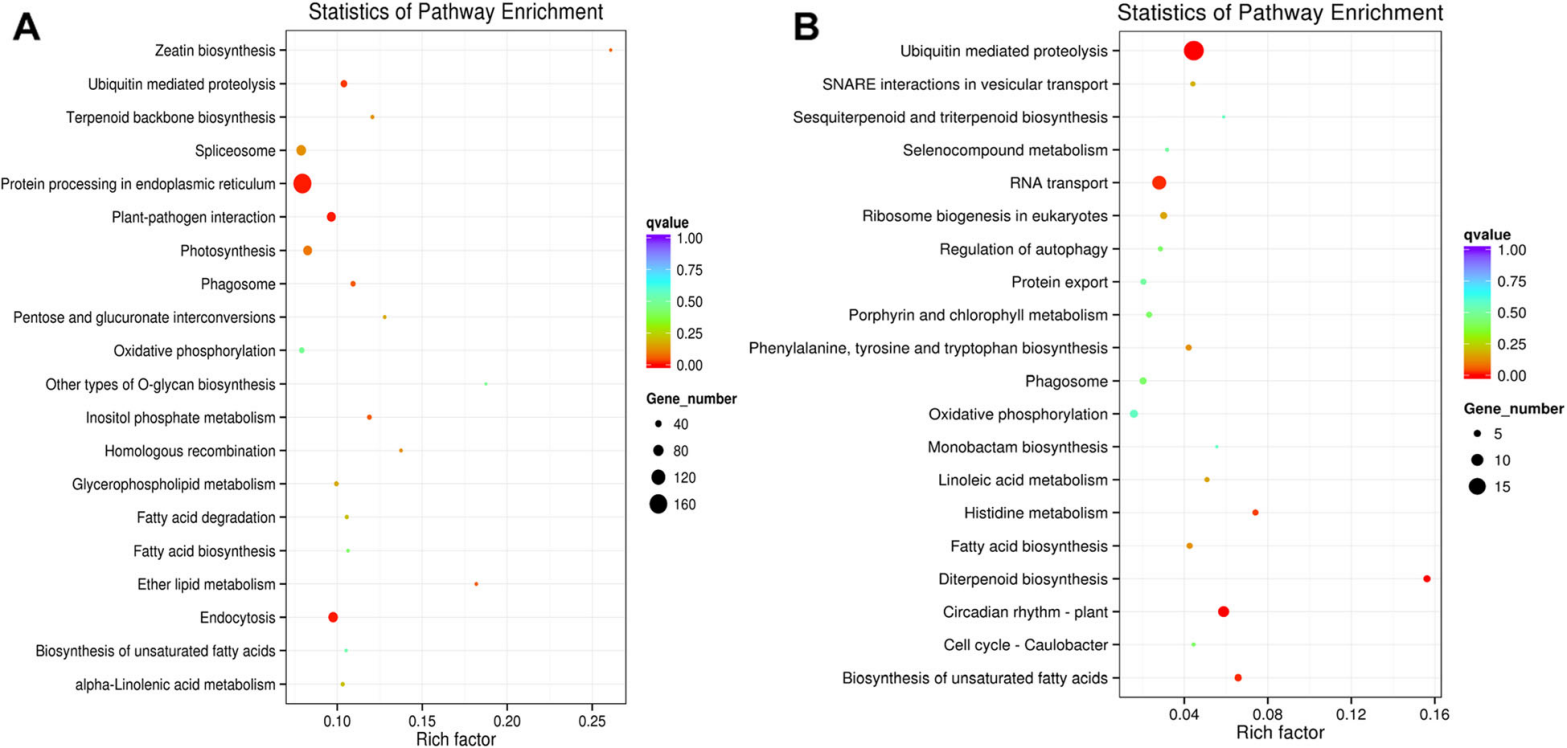
Scatter diagram of miRNA targeted genes on KEGG pathways. **a**, genes identified in ‘CH3’ and ‘CH5’. **b**, genes identified in ‘Tillering’ and ‘Pre-tillering’

Results showed that the oxidation-reduction process in the biological process (BP) and membrane protein complex in the cellular component (CC) were the most enriched GO terms with 212 (10.4%) and 112 (5.5%) genes, respectively. Between Tillering and Pre-tillering, 1140 target genes of miRNA were identified and categorized into 42 GO functional subcategories which were fewer than between Ch-3 and Ch-5. Molecular function (MF) showed the highest enrichment in Tillering and Pre-tillering, and oxidoreductase activity (32, 9.58%), coenzyme binding (19, 5.69%) and cofactor binding (21, 6.29%) were the most abundant GO terms, which were different with the dominant GO terms enriched in BP and CC in Ch-3 and Ch-5. However, we found some co-enriched target genes in both Ch-3/Ch-5 and Tillering/Pre-tillering groups using KEGG analysis (Fig. 5; Additional file 14). About 689 target genes were mapped into 20 KEGG pathways in the Ch-3/Ch-5 group, while 100 genes were mapped into 20 KEGG pathways in the Tillering/Pre-tillering group. Fortunately, five KEGG pathways such as “ubiquitin-mediated proteolysis”, “phagosome”, “fatty acid biosynthesis”, “oxidative phosphorylation” and “biosynthesis of unsaturated fatty acids” showed co-enrichment in both Ch-3/Ch-5 and Tillering/Pre-tillering groups. The most co-enrichment of miRNA target genes were detected in the “ubiquitin-mediated proteolysis” and “oxidative phosphorylation”, suggesting that these two pathways may play a great role in controlling tillering development in the grass.

### Validation of miRNA expression patterns by qRT-PCR

**Fig. 6:**
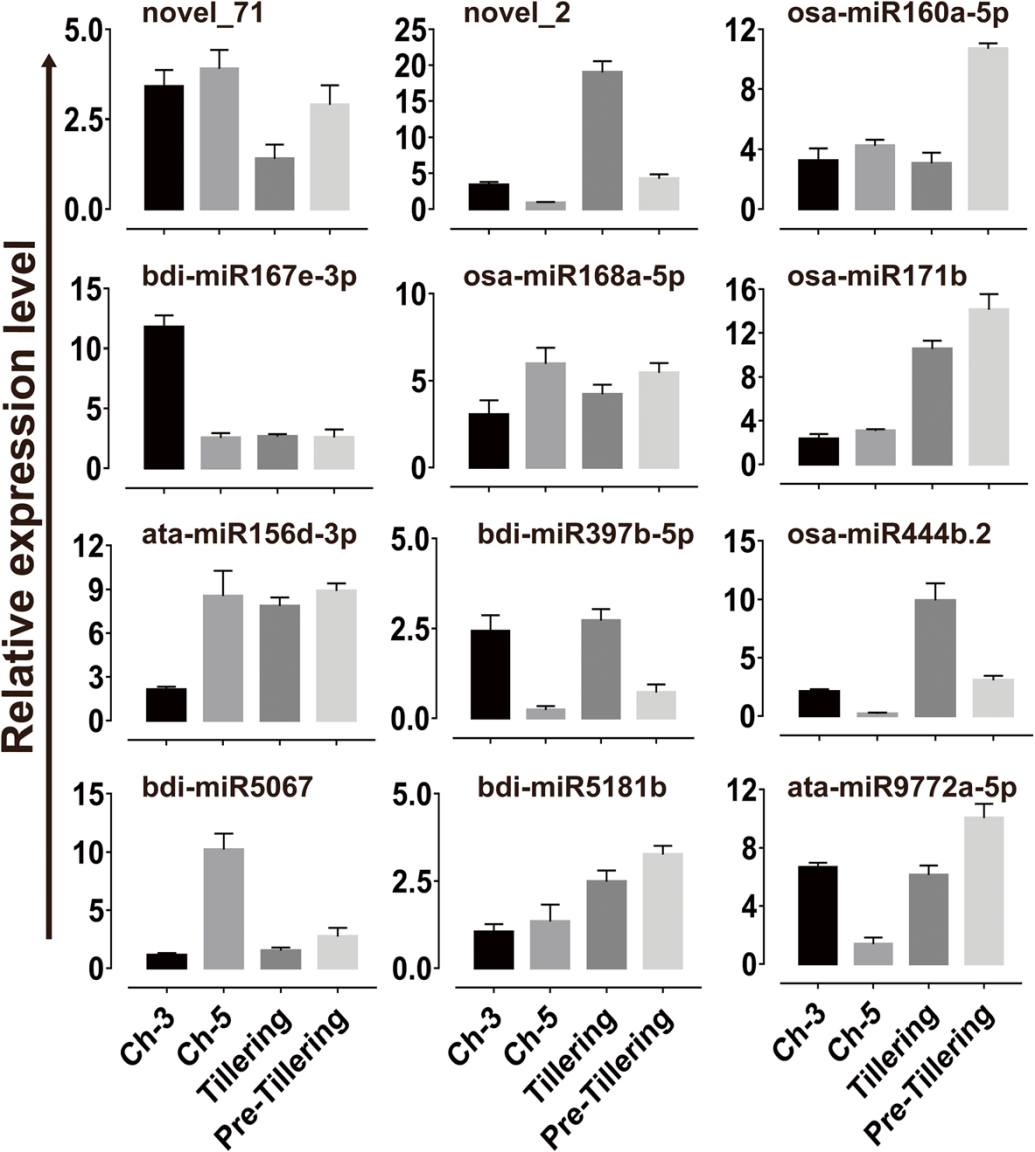
Expression analysis of 12 randomly selected miRNAs by stem–loop qRT-PCR. 18 s rRNA gene was used as the reference gene. Error bars represent the SE for three independent experiments, and three technical replicates were analyzed. Vertical bars indicated LSD values where significant difference were detected (*P* < 0.05). ‘Ch-3’ with high tiller productive rate and ‘Ch-5’ with few tillers were collected during tillering-stage tall fescue. Pre-tillering and Tillering indicates samples were collected before and after tiller producing stage

To validate the small RNA sequencing results involved in tillering development, we analyzed the expression level of twelve miRNAs using Stem-Loop qRT-PCR. Twelve miRNAs were randomly selected including two novel miRNAs, seven conserved miRNAs and three non-conserved miRNAs. As shown in Fig. 6, miRNA novel-2, osa-miR444b.2 and bdi-miR397b-5p were co-up-regulated, while bdi-miR5067 showed co-down-regulated in the Ch-3 and Tillering grass compared with the Ch-5 and Pre-tillering grass, which was in line with miRNA sequencing results. The abundance of miRNA novel-2, osa-miR160a-5p and osa-miR171b were significantly increased in the Pre-tillering grass compared to Tillering ones, but the expression did not change between Ch-3 and Ch-5 grasses. However, bdi-miR167e-3p exhibited higher expression level but ata-miR156d-3p and osa-miR168a-5p showed lower expression levels in Ch-3 than that in Ch-5, while their expression level was not changed between Tillering and Pre-tillering grass. The transcript abundance of bdi-miR5181b had no difference between both tall fescue genotypes Ch-3 and Ch-5 and between Tillering and Pre-tillering grass. Interestingly, ata-miR9772a-5p showed increased transcript abundance in Ch-3 than that in Ch-5, but had a lower expression level in the Tillering grass compared with the Pre-tillering grass. Additionally, the correlation between small RNA sequencing and qRT-PCR results were evaluated in terms of transcript abundance. As shown in Additional file 15, the qRT-PCR measurements were moderately correlated with miRNA profiles revealed by Illumina sequencing (y = 0.0085x + 2.4609; R^2^ = 0.5431; *P* < 0.001), indicating that the small RNA sequencing data was accurate and effective.

## Discussion

Tiller number is considered as the main agronomic trait for grass and numerous studies have been focused on elucidating molecular mechanisms of tiller development. Nevertheless, studies to identify miRNA-genes modules controlling the tillering process are more obscure. The current study presented the genome-wide sRNA profiles of two tall fescue genotypes contrasting for tillering production rate and the tissue at both vegetative and tillering stages based on full-length transcriptome data. A total of 222 million high-quality clean reads were generated and 208 miRNAs were identified in tall fescue. Among them, 60 miRNAs showed different expression patterns between the two tall fescue genotypes with a different tillering rate or from the tissues at different tillering development stages. Furthermore, we initially detected 14 miRNAs co-up-regulated and 4 miRNAs co-down-regulated in both Ch-3/Ch-5 and Tillering/Pre-tillering groups, suggesting the potential key regulators controlling tillering development in cool-season turfgrass.

Comparing different genotypes or tiller development stages, previous studies have explored vital regulatory elements involved in molecular mechanisms of tillering [26–29]. Alam et al. [26] and Saxena et al. [27] reported that environmental factors such as temperature-induced tillering variation among different sorghum genotypes and tall fescue genotypes. Matts et al. [29] performed miRNA expression profiling in the emerging tillers from the young plants and inflorescence from the adult plants in switchgrass. Li, et al. [28] analyzed miRNA regulation networks of tillering with tiller primordia, stem tips, and young spikes in one high yield wheat cultivar ‘Guomai 301’. However, these studies just analyzed tillering in different genotypes or at different tiller development stages individually, and few studies have coupled genotypes variation with tiller development stages to verify tillering regulatory-model. Here, Ch-3 was undergoing vegetative growth produced 122 tillers, while Ch-5 begun heading growth and only had 9 tillers after 2 months of establishment, indicating that these two tall fescue genotypes had a remarkable variation in tillering development. Pre-tillering samples were collected before tillering and Tillering samples were collected after tillering. The analysis by coupling Ch-3, Ch-5, Pre-tillering, and Tillering samples will provide one significant tillering regulatory-model without the background interference of genotypic variation and tissue development changes in tall fescue.

In this study, 222 million reads were obtained, which were much higher than our previous small RNA sequencing analysis (six million reads) in tall fescue in response to heat stress [25]. Based on the full-length transcriptome data of tall fescue and miRBase21 database annotation, 3051 miRNA precursors were identified in Ch-3, Ch-5, Pre-tillering and Tillering samples. Finally, 208 miRNAs including 148 known and 60 novel miRNAs in tall fescue were obtained by blastx searching against mature miRNAs from *Brachypodium distachyon*, *Oryza sativa*, *Zea mays*, *Hordeum vulgare*, *Aegilops tauschii* and *Festuca arundinacea*, which were more than the tillering-responsive small RNAs (32 miRNAs) identified in switchgrass through a genome-wide analysis [29]. In this study, the length of the miRNAs ranged from 18 to 24 nt, which was consistent with the previous reports [25, 30]. In wheat, 420 miRNAs were obtained to analyze the regulation networks of tillering [28]. The miRNAs identified in this study were fewer than those found in wheat probably because wheat has a reference genome while tall fescue lacks genome information. In our previous work, we carried out transcriptome analysis in tall fescue and small RNA analysis and identified temperature-responsive small RNAs (including the full-length transcriptome, data unpublish). Both RNA-Seq and small RNA analyses provided rich information for the understanding of the molecular mechanism of tillering development or heat stress response in tall fescue.

Previous works have proven that miRNAs play crucial roles in controlling tiller number in plant, mainly including Osa-miR156–SPLs, Osa-miR393–OsTIR1/OsAFB2, Osa-miR444–OsMADS23/27a/57, Osa-miR529–SPL14/17and Osa-miR528 [11, 23]. In rice, miR156 could repress OsSPL14 functions by cleaves its mRNA, and miR156-OE plants resulted in rapid tiller initiation [21]. Furthermore, tiller number decreased when OsSPL14 mutated at the OsmiR156-targeted site [31]. *OsMADS57* expression was significantly reduced in osa-miR444a-overexpressing rice, which showed fewer tillers than did WT [22]. In miR393-overexpressing rice, the expression of *OsAUX1* and*OsTB1* were down-regulated and tiller number increased [19]. In switchgrass, transgenic plants overexpressing miR393 also showed more tillers and higher biomass yield in greenhouse and field tests [18]. However, Osa-miR393a overexpressing creeping bentgrass had fewer and longer tillers compared with WT [32]. Wang et al. [33] reported that overexpressing Osa-miR529 in rice inhibited *SPL14/17* and enhanced the tiller number. In creeping bentgrass, miR528-overexpressing plants display increased tiller number, and upright growth [23]. Here, we identified 18 miRNAs involved in tall fescue tiller development. Among them, 14 miRNAs displayed increased abundance in both Ch-3 with high productive tiller rate and Tillering plants compared with that in few tillers Ch-5 and Pre-tillering plants, which were positively correlated with tiller development in tall fescue. The osa-miR156a was included within these 14 miRNAs. And more than two times abundance of osa-miR156a were measured in Ch-3 relative to Ch-5 plants, and tillering plants also obtained higher expression level of osa-miR156a than in Pre-tillering plants. This is consistent with the result that miR156-OE plants had greater abundance than WT plants [21, 31]. The expression level of zma-miR528a-3p increased in Ch-3 and Tillering plants and was positive with tiller development, which was is consistent with the results that miR156-OE increased the tiller number in creeping bentgrass [23]. On the contrary, the expression levels of another four miRNAs was significantly decreased in Ch-3 and Tillering plants relative to Ch-5 and Pre-tillering plants, which showed one negative relationship with tiller development in tall fescue. Among them, osa-miR444b.2 was down-regulated in Ch-3 and Tillering plants, which was opposite to the result in osa-miR444a-overexpressing rice with fewer tillers [22].

Besides, osa-miR156a, zma-miR528a-3p and osa-miR444b.2 and the rest 15 miRNAs are the novel regulators involved in tiller development in plants. Bdi-miR160f and osa-miR408-3p belonged to miR160 and miR408 family, respectively, which was recognized as the most conserved miRNA family and identified in the common ancestor of all embryophytes [34]. Meanwhile, osa-miR408-3p was up-regulated in Ch-3 and Tillering plants and was positive with tiller development, and also involved in abiotic stress responses in Arabidopsis [35]. However, bdi-miR160f was negative with tillering development in tall fescue, which was reported in tomato involving auxin-mediated ovary patterning as well as floral organ [36]. In this study, bdi-miR167e-3p, bdi-miR397b-5p, hvu-miR397a, osa-miR397a, osa-miR397b, and osa-miR394 were associated with tiller development and belonged to miR167, miR397 and miR394 family, respectively, which were acquired in the common ancestor of all spermatophytes [34]. In rice and Arabidopsis, miR167 was found to be involved in floral transition and organ, miR394 could control leaf development and miR397 could mediate seed development [11], suggesting they may have the potential of regulating tiller development in grass plant. Here, we found that bdi-miR167e-3p targets two kinase proteins EIF2AK4 and IRAK4, and osa-miR397a targets auxin response factor 5, indicating grass tillering may be relative to miRNA-kinases/ phytohormone signalling. Ata-miR5168-5p and zma-miR1432-5p belonged to miR5168 and miR1432 family which was conserved in monocots. Ata-miR5168-5p showed higher expression level in Ch-3 and Tillering compared with Ch-5 and Pre-tillering plants and was positive with tiller development. Recently, few works involved in miR5168 mediating plant stress response or development. On the contrary, zma-miR1432-5p was negative with Tillering in tall fescue, which inhibited *OsACOT* and played an important role in rice grain filling [37]. Additionally, five novel miRNAs including novel_13, novel_18, novel_2, novel_22, and novel_23 involved in tillering were identified in tall fescue, which may play unique roles in the tiller development of grass plants.

miRNAs control tiller development by miRNA-mRNA modules in plants. Here, we obtained a total of 28,927 potential target genes for all identified miRNAs, which was more than the target genes number (790) found in tall fescue in response to heat stress [25]. GO enrichment analysis showed that, oxidation-reduction process (212, 10.4%) in the BP category and membrane protein complex (112, 5.5%) in the CC category were the most enriched GO terms in Ch-3 and Ch-5. However, in Tillering and Pre-tillering plants, MF was the dominant category with the highest enriched GO terms including oxidoreductase activity (32, 9.58%), coenzyme binding (19, 5.69%) and cofactor binding (21, 6.29%). In the KEGG pathways enrichment analysis, five pathways including “ubiquitin-mediated proteolysis”, “phagosome”, “fatty acid biosynthesis”, “oxidative phosphorylation”, and “biosynthesis of unsaturated fatty acids” were co-enriched in both Ch-3/Ch-5 and Tillering/Pre-tillering groups, among which “ubiquitin-mediated proteolysis” and “oxidative phosphorylation” were the most co-enriched pathways of miRNA target genes. Most of the target genes of 18 tall fescue tillering relative miRNAs were mapped into these five KEGG pathways. Further studies will be conducted to ascertain miRNA-gene pathways involved in tiller development in tall fescue.

## Conclusions

This study demonstrated the first genome-wide small RNAs profiles dataset in tall fescue involved in tiller development. More than 222 million reads were generated and a total of 208 miRNAs were identified. 18 miRNAs were directly related to the tall fescue tillering process, among which 15 miRNAs were novel regulators involved in tiller development in tall fescue plants. A total of 28,927 potential target genes were identified for all miRNAs. Most of the 212 target genes of tillering related 18 miRNAs were assigned into “ubiquitin-mediated proteolysis”, “phagosome”, “fatty acid biosynthesis”, “oxidative phosphorylation”, and “biosynthesis of unsaturated fatty acids” KEGG pathways. Overall, this study reveals novel insights into the molecular mechanisms of miRNA-genes that related to tiller development in tall fescue.

## Methods

### Plant materials, growth conditions and experimental design

Healthy and single clonal plants of two tall fescue genotypes, ‘CH-3’ with high tiller productive rate and ‘CH-5’ with few tillers were collected from turfgrass field plots at Wuhan Botanical Garden, Chinese Academy of Sciences, Wu Han, Hubei, China, and were cultivated in a greenhouse under natural sunlight at 22/18 °C (day/night) with 87% relative humidity and wind speed of 0.8 m/s. After 2 months of establishment, root-stem junction tissues of ‘CH-3’ and ‘CH-5’ during tillering-stage were collected and froze immediately with liquid nitrogen, and then stored at − 80 °C. Seeds of tall fescue (cv. ‘Houndog V’) were germinated in filter paper and transplanted to plastic pots (13 cm diameter, 11 cm deep) filled with a mixture of sand and peat soil (1/1, v/v) in the same greenhouse above. When seedlings reached two fully-expanded leaf and one unexpanded leaf (2.5-leaf stage) no tiller produced, root-stem junction tissues as pre-tillering group samples were collected. When seedlings with 4.5-leaf stage were tillering, root-stem junction tissues as tillering group samples were collected. Removing root and stem tissues from the root-stem junction sites, and just leaving their junction tissues where the tillers buds began to grow for subsequent analysis. All samples were cleaned with distilled water and stored at − 80 °C. At least seven plants from each CH-3, CH-5, pre-tillering and tillering group sample were pooled for each biological replicate, and three biological replicates were performed.

### RNA extraction, sRNAs library construction and sequencing

Total RNA was extracted from tall fescue root-stem junction tissues using Trizol reagent (Invitrogen, Carlsbad, CA). The concentration and quality of the total RNA was determined by a NanoDrop 8000 spectrophotometer (NanoDrop, Wilmington, DE) and checked by Agilent Bioanalyzer 2100 system. Only high integrity RNA was used for the sequencing analysis. 3 μg of total RNA for each sample was used to construct a sRNAs library using NEBNext® Multiplex Small RNA Library Prep Set for Illumina® (NEB, USA.) following manufacturer’s recommendations. Library quality was assessed on the Agilent Bioanalyzer 2100 system by DNA High Sensitivity Chips. When RNA Integrity Number (RIN) is more than 7 and the library effective concentration is more than 2 nM, the pooled sRNA libraries were sequenced on the Illumina HiSeq 2500 at the Novogene Co., Ltd. (Beijing, China) to produce 50-bp single-end reads.

### SRNAs sequencing data processing

The raw data were filtered through custom perl and python scripts to remove containing more than 10% N, with 5′ adapter contaminants, without 3′ adapter or the insert tag, containing probably poly A or T or G or C reads and low quality reads. Then 18–30 nt clean reads were mapped to reference sequence by Bowtie [38]. Bowtie indexes the reference sequence through the Burrows-Wheeler transform and the full-text minute-space index [38]. And memory-efficient alignment program was used for aligning short DNA sequence reads to large reference without mismatch to analyze their expression and distribution on the reference. Here, we chose full-length transcriptome data of tall fescue (data unpublish) as the reference sequence. Reads matched to protein-coding genes, repeat sequences, rRNA, tRNA, snRNA, snoRNA and sRNAs tags were removed. To identify known miRNAs and draw their secondary structures, we aligned all reads against miRNA registered in miRbase 20.0 using mirdeep2 [39] and srna-tools-cli (http://srnatools.cmp.uea.ac.uk). For novel miRNA identification, the available software miREvo [40] and mirdeep2 [39] were integrated to predict miRNA through exploring the secondary hairpin structure.

### Differential expression analysis of miRNAs

Expression level for each identified miRNA was estimated by TPM (transcript per million) through the following the normalization formula: Normalized expression = Mapped readcount/Total reads*1000000 [41]. Differential expression analysis of two groups was performed using the DESeq R package (version 2.14, http://www.bioconductor. org/packages/release/bioc/html/DESeq.html) with a threshold of corrected *p*-value < 0.05 and the absolute value of fold-change > 2.

### Target genes prediction of miRNAs, and GO and KEGG enrichment analysis

Predicting the target gene of miRNA was performed by psRobot_tar in psRobot [42]. Gene Ontology (GO) enrichment analysis for the target gene candidates of differentially expressed miRNAs was performed using GOseq based Wallenius non-central hyper-geometric distribution. KOBAS software was used to test the statistical enrichment of the target gene candidates in Kyoto Encyclopedia of Genes and Genomes (KEGG) pathways [43].

### Quantitative RT-PCR analysis

Stem-Loop quantitative reverse transcription PCR (qRT-PCR) was carried out to examine the expression level of miRNAs. The stem–loop qRT-PCR primers were designed according to Varkonyi-Gasic et al. [44] shown in Additional file 16. Stem–loop RT reactions were performed at 16 °C for 30 min, followed by 42 °C for 30 min, 85 °C for 5 min in 20 μL reaction systems contained 2 μg of RNA samples, 1 μL denatured stem-loop RT primer, 0.5 μL 10 mM dNTP mix, 4 μL 5X First-Strand buffer, 2 μL 0.1 M DTT, 11.15 μL nuclease-free water, 0.1 μL RNaseOUT (40 U/μL), 0.25 μL SuperScript III RT (200 U/μL) (Invitrogen, USA). 18S rRNA was used as the internal control. qRT-PCR was performed on ABI StepOne Plus Real-Time PCR system (Applied Biosystems, Foster City, CA) and SYBR Green Real-Time PCR Master Mix (Toyobo, Osaka, Japan) in 20 μL reactions according to the manufacturer’s protocol. The relative expression levels of miRNAs were measured using the 2^-△△Ct^ method.

### Statistical analysis

The data obtained from qRT-PCR were expressed as the mean ± standard deviation. The mean separation was performed with Fisher’s least significant difference test at *P* < 0.05 using the Statistical Analysis System (SAS 9.0 for windows, SAS Institute Inc., Cary, NC). Three biological replicates were prepared for each sample.

## Supplementary information

**Additional file 1.** Maping rate of unique reads to full-length transcriptome data in tall fescue.

**Additional file 2.** Length distribution of miRNAs in two tall fescue genotypes in Ch-3, Ch-5, Pre-tillering and Tillering smaples.

**Additional file 3.** The list of known miRNAs identified in tall fescue.

**Additional file 4.** miRNAs family analysis in tall fescue.

**Additional file 5.** The list of novel miRNAs identified in tall fescue.

**Additional file 6.** The first base preference in 18 ~ 30-nt sRNAs for identified novel miRNAs.

**Additional file 7.** Differential expression analysis of miRNAs used for linkage hierarchical clustering analysis (all data were the TPM value of miRNAs).

**Additional file 8.** Differential expression of miRNAs between Ch-3 and Ch-5 tall fescue.

**Additional file 9.** Differential expression of miRNAs between Pre-tillering and Tillering tall fescue.

**Additional file 10.** Venn diagrams showing co-up-regulated and co-down-regulated miRNAs involved in tall fescue tillering.

**Additional file 11 **Expression analysis of 6 randomly selected miRNAs relative to tillering by stem–loop qRT-PCR. 18 s rRNA gene was used as the reference gene. Error bars represent the SE for three independent experiments, and three technical replicates were analyzed. Vertical bars indicated LSD values where significant difference were detected (*P* < 0.05). ‘Ch-3’ with high tiller productive rate and ‘Ch-5’ with few tillers were collected during tillering-stage tall fescue. Pre-tillering and Tillering indicates samples were collected before and after tiller producing stage.

**Additional file 12.** Prediction of miRNA target genes in Ch-3, Ch-5, Tillering and Pre-tillering samples.

**Additional file 13.** The details of Go enrichment of miRNA target genes.

**Additional file 14.** The list of KEGG pathway for miRNA target genes at Ch-3/Ch-5 and Tillering/Pre-tillering groups.

**Additional file 15.** Correlations of expression level analyzed by small RNA-Sequencing (x axis) with data obtained using qRT-PCR (y axis).

**Additional file 16.** All the primers used in this study.

## Data Availability

The raw sequences for *Festuca arundinacea* Schreb. ‘Ch-3’, ‘Ch-5’, Pre-tillering and Tillering samples have been deposited in the NCBI Sequence Read Archive (SRA) under BioProject PRJNA629066. Accession numbers for the miRNA sequencing are SRR11640118, SRR11640119, SRR11640120 and SRR11640121 (https://dataview.ncbi.nlm.nih.gov/object/PRJNA629066).

## Abbreviations

miRNAs: MicroRNAs
nt: nucleotides
MIRs: miRNA genes
pri-miRNAs: precursor miRNAs
Pol II: polymerase II
DCL1: Dicer-like protein 1
HYL1: HYPONASTIC LEAVES1
SE: SERRATE
HEN1: HUA ENHANCER 1
AGO1: ARGONAUTE 1
miRISC: miRNA-induced silencing complex
UTRs: untranslated regions
qRT-PCR: quantitative reverse transcription PCR
KEGG: Kyoto encyclopedia of genes and genomes
sRNAs: small RNAs
snoRNAs: small nucleolar RNAs
snRNAs: small nuclear RNAs
GO: Gene ontology
