# Supplementary figures and images for: Genome-wide small RNA profiling reveals tiller development in tall fescue (Festuca arundinacea Schreb)
Source: BMC Genomics. 2020 Oct 6;21:696. doi: 10.1186/s12864-020-07103-x (PMC7539525; doi:10.1186/s12864-020-07103-x)

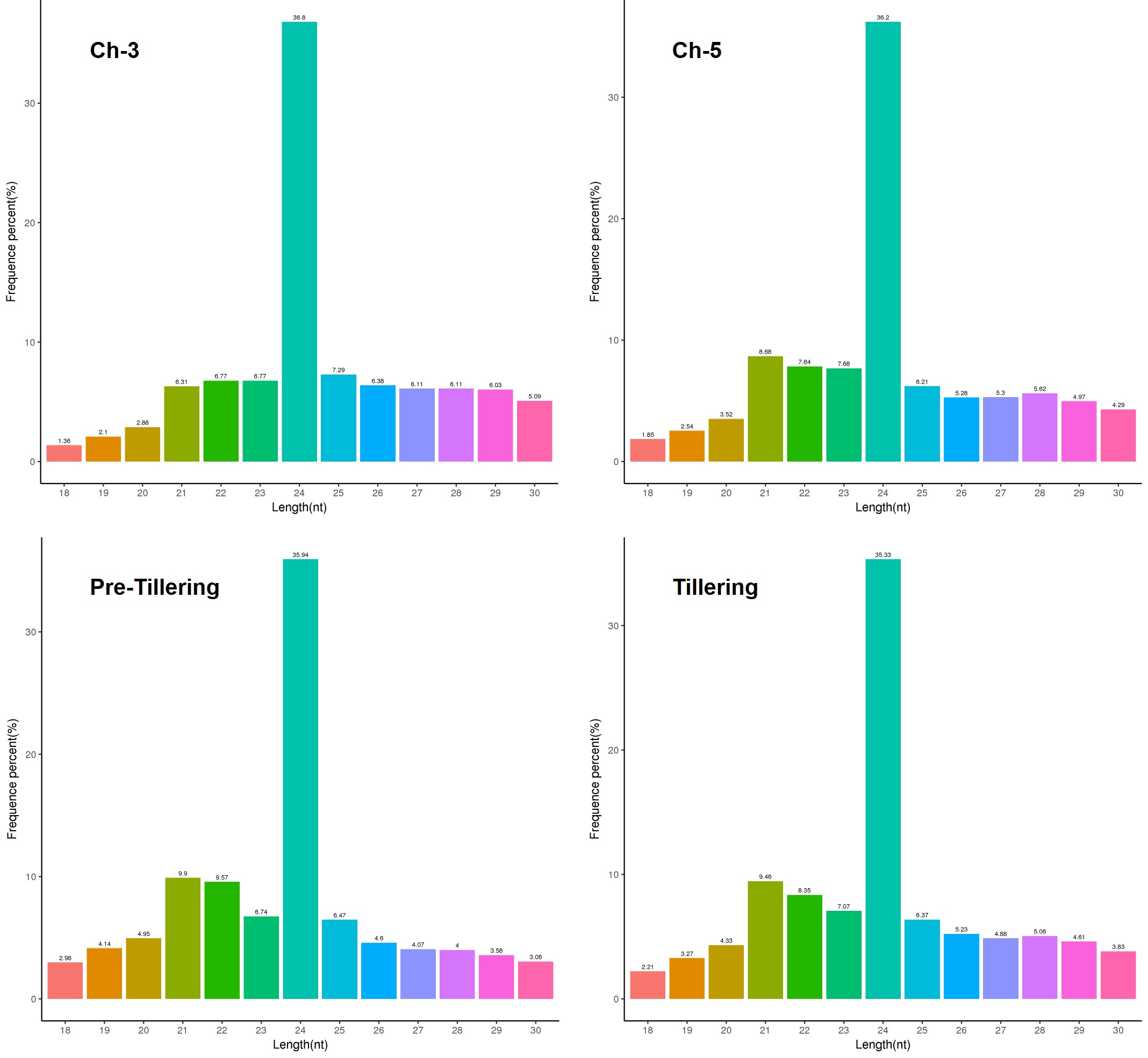

Supplement: Supplementary file 2 — Additional file 2. Length distribution of miRNAs in two tall fescue genotypes in Ch-3, Ch-5, Pre-tillering and Tillering smaples. [file 12864_2020_7103_MOESM2_ESM.jpg]

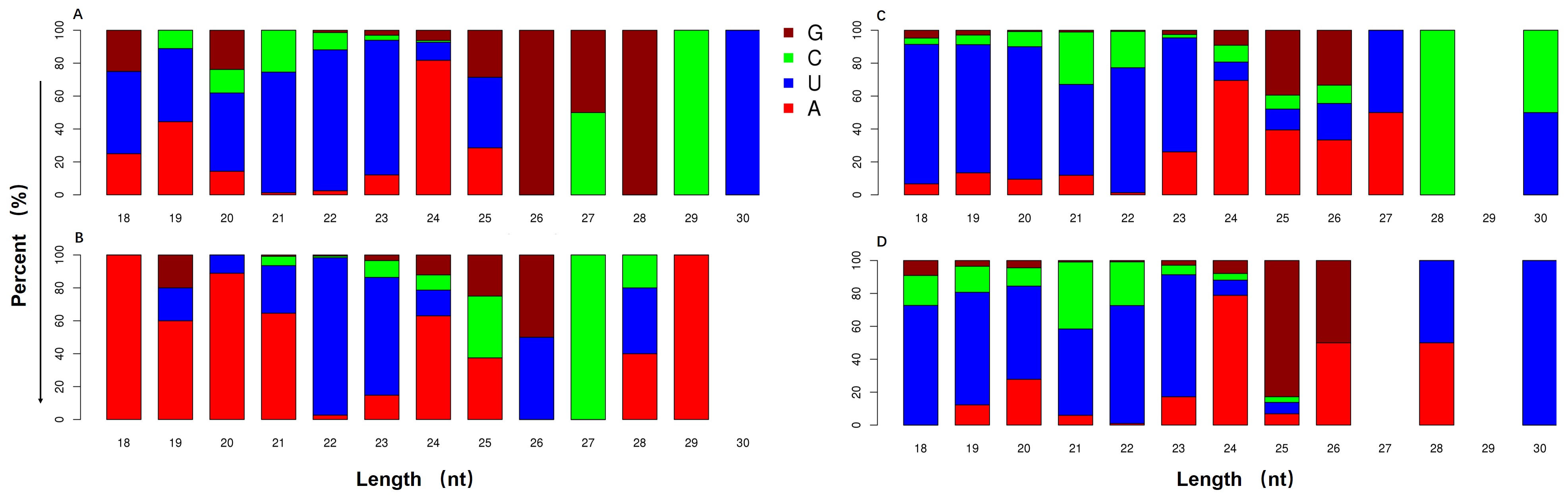

Supplement: Supplementary file 6 — Additional file 6. The first base preference in 18 ~ 30-nt sRNAs for identified novel miRNAs. [file 12864_2020_7103_MOESM6_ESM.jpg]

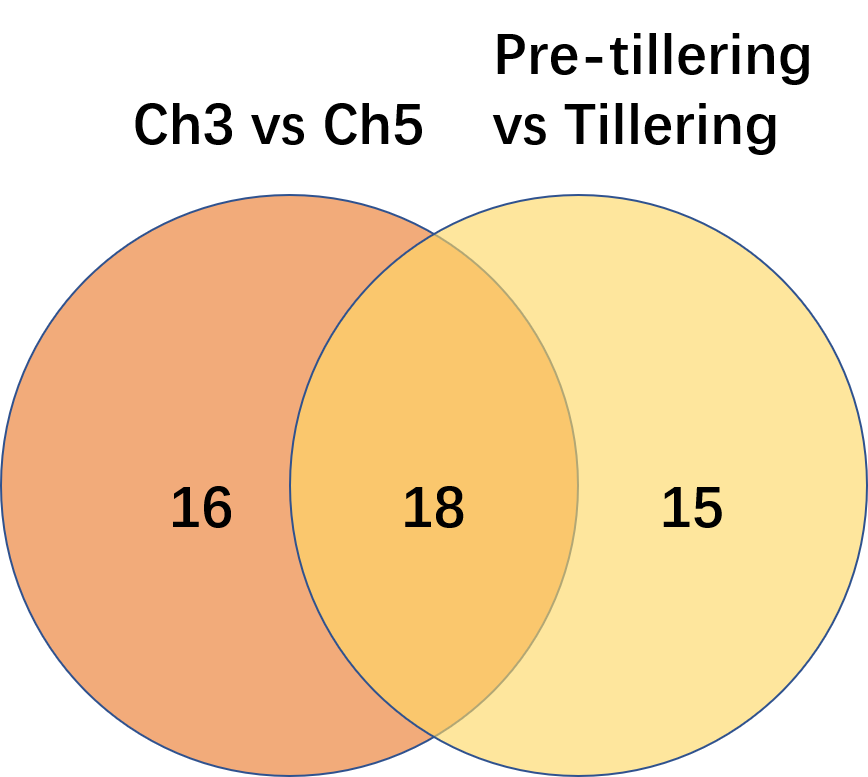

Supplement: Supplementary file 10 — Additional file 10. Venn diagrams showing co-up-regulated and co-down-regulated miRNAs involved in tall fescue tillering. [file 12864_2020_7103_MOESM10_ESM.tif]

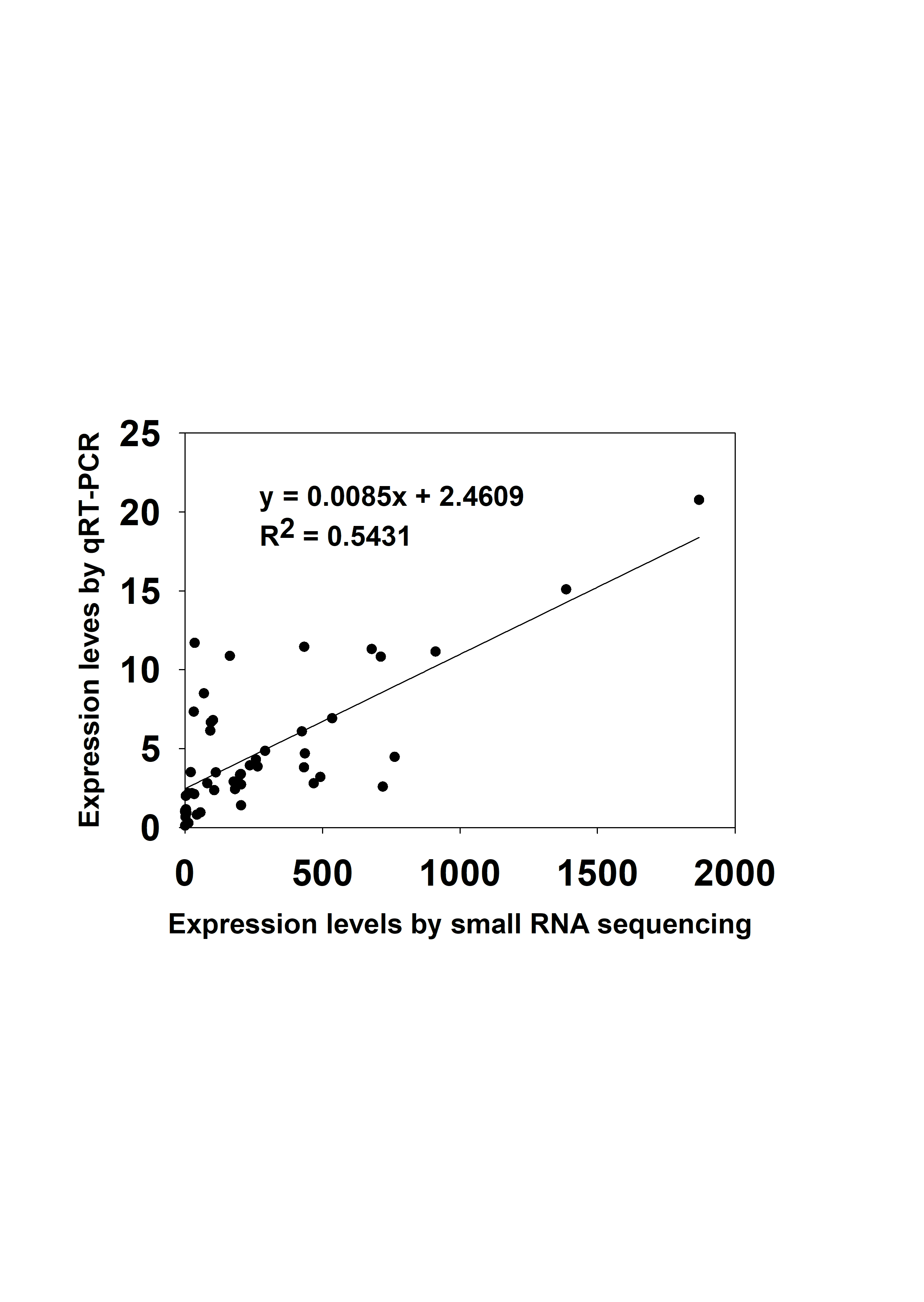

Supplement: Supplementary file 15 — Additional file 15. Correlations of expression level analyzed by small RNA-Sequencing (x axis) with data obtained using qRT-PCR (y axis). [file 12864_2020_7103_MOESM15_ESM.tif]
